# Tracking Topological and Electronic Effects on the Folding and Stability of Guanine-Deficient RNA G-Quadruplexes, Engineered with a New Computational Tool for De Novo Quadruplex Folding

**DOI:** 10.3390/ijms231910990

**Published:** 2022-09-20

**Authors:** Yavuz Burak Göç, Jakub Poziemski, Weronika Smolińska, Dominik Suwała, Grzegorz Wieczorek, Dorota Niedzialek

**Affiliations:** 1 Institute of Biochemistry and Biophysics, Polish Academy of Sciences, Pawińskiego 5a, 02-106 Warsaw, Poland or; 2 Faculty of Chemistry, Biological & Chemical Research Center, University of Warsaw, Pasteura 1, 02-093 Warsaw, Poland; 3 Faculty of Chemical and Process Engineering, Warsaw University of Technology, Waryńskiego 1, 00-645 Warsaw, Poland; 4 Faculty of Physics, University of Warsaw, Pasteura 5, 02-093 Warsaw, Poland; 5 Molecure SA, Żwirki i Wigury 101, 02-089 Warsaw, Poland

**Keywords:** RNA G-quadruplex folding, RNA—cation interactions, valence tautomerism, de novo RNA modelling, RNA G-quadruplex stabilization

## Abstract

The initial aim of this work was to elucidate the mutual influence of different single-stranded segments (loops and caps) on the thermodynamic stability of RNA G-quadruplexes. To this end, we used a new NAB-GQ-builder software program, to construct dozens of two-tetrad G-quadruplex topologies, based on a designed library of sequences. Then, to probe the sequence–morphology–stability relationships of the designed topologies, we performed molecular dynamics simulations. Their results provide guidance for the design of G-quadruplexes with balanced structures, and in turn programmable physicochemical properties for applications as biomaterials. Moreover, by comparative examinations of the single-stranded segments of three oncogene promoter G-quadruplexes, we assess their druggability potential for future therapeutic strategies. Finally, on the basis of a thorough analysis at the quantum mechanical level of theory on a series of guanine assemblies, we demonstrate how a valence tautomerism, triggered by a coordination of cations, initiates the process of G-quadruplex folding, and we propose a sequential folding mechanism, otherwise dictated by the cancellation of the dipole moments on guanines.

## 1. Introduction

More and more scientific reports indicate the wide occurrence of guanine quadruplexes (GQs) across the transcriptome in various regions of mRNA, non-coding RNAs and telomeric 3${}^{\prime }$overhangs, being involved in replication and genomic instability [1,2,3]. The GQ-forming sequences are overrepresented in oncogene promoter regions such as *hTERT*, *MYC*, *BCL2*, *c-KIT* and *KRAS* [4]. The latter is the most frequently mutated oncogene in human cancers and the Kristen rat sarcoma viral oncogene homologue (*KRAS*) mutation is commonly associated with poor prognosis and resistance to therapy. For this reason, quadruplexes have become emerging potential therapeutic targets, as the transcriptional repression of oncogenes through the stabilization of their GQ topologies has become a novel anticancer strategy.

**Figure 1:**
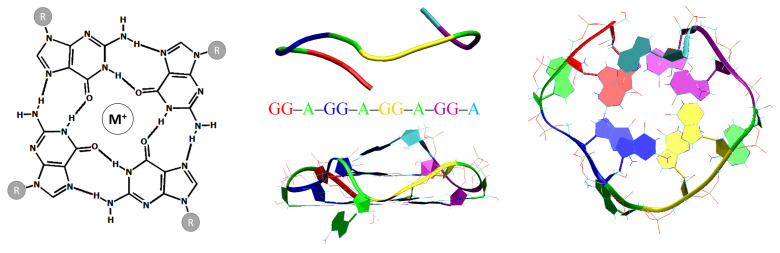
**Left** panel: Schematic representation of a G-tetrad with a coordinated metal cation. **Central** and **right** panels: The PDB ID 2RQJ structure that was used as a reference for the investigated series of intramolecular RNA two-tetrad G-quadruplex systems. Loops and caps are coloured green and cyan, respectively.

The guanine quadruplexes are non-canonical four-stranded nucleic acid helices (i.e., DNA or RNA molecules), assembled from repeated short sequences of guanines in the presence of (preferably monovalent) cations [5]. As presented in Figure 1, GQ secondary structures are formed of at least two stacks of cyclic arrangements of four Hoogsteen hydrogen-bonded co-planar guanosine residues (G-tetrads). Each guanine in the tetrad acts both as an acceptor and a donor of two hydrogen bonds. The spatial proximity of the four carbonyl oxygens in the G-tetrad is unique, with their lone electron pairs in a very close approach, which produces a significant electrostatic repulsion of negative charges and, therefore, represents an excellent location for the coordination of dehydrated cations. Quadruplexes can be formed intramolecularly or by the association of two or four nucleic acid molecules, and some of them also have the tendency to oligomerize. The tracks of the stacked guanines (G-strands) are connected to the sugar-phosphodiester backbone(s) via glycosidic bonds, which can adopt the anti or syn geometries, depending on the relative orientation of the four G-strands (regarding their 5${}^{\prime }$ to 3${}^{\prime }$ polarity): parallel (the four strands share the same polarity), antiparallel (two strands one way, the other two the opposite way; these pairs correspond to two adjacent or diagonally opposed G-strands, resulting in very different geometries) or hybrid (three strands sharing the same polarity and the last one having the opposite). The parallel topologies contain almost exclusively anti guanines, whereas mixtures of syn and anti are observed in antiparallel and hybrid structures. Finally, the single-stranded sections that link G-tetrads are called loops and can also adopt diverse geometries: lateral (edgewise), diagonal and double-chain reversal (propeller) [5].

The length and composition of the loop motifs are major determinants of the thermal stability and folding topology of quadruplexes [6,7,8]. For example, the shortest, single-nucleotide loops intrinsically form the propeller geometry, which corresponds to the parallel orientation of the four G-strands that are inherent in RNA G-quadruplexes. The combination of different loop geometries, orientations of G-strands and molecularities determines the polymorphism of GQs [9], with 26 theoretically possible G-quadruplex topologies for nucleic acids, among which only a few have been observed in vitro [10,11,12]. Ultimately, the topology of a G-quadruplex depends on its precise nucleotide sequence, which can provide additional stabilizing interactions via pi-stacking, hydrogen bonding, solvation and cation coordination, but also on buffer conditions, i.e., the types of cations in the solvent.

**Figure 2:**
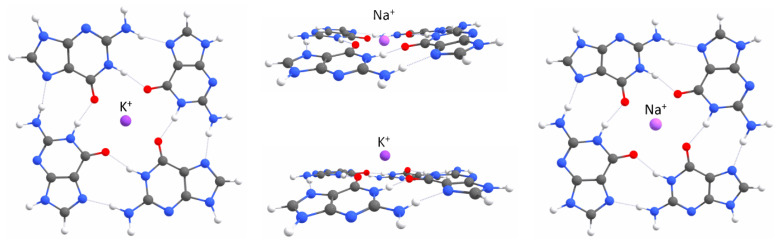
G-tetrads stabilized by potassium (**left**) and sodium (**right**) ions, calculated at the QM level of theory. The **central** panel presents different positions of K${}^{+}$ and Na${}^{+}$ cations with respect to the plane of G-tetrads.

G-quadruplex formation has been demonstrated in the presence of monovalent and divalent cations, with the structural stabilization following this general trend: Sr${}^{2+}$ > K${}^{+}$ > Ca${}^{2+}$ > NH${}^{+}$ > Na${}^{+}$ > Rb${}^{+}$ > Mg${}^{2+}$ > Li${}^{+}$ > Cs${}^{+}$ [13,14]. Still, the majority of physical studies reported for G-quadruplexes have been conducted in solutions containing Na${}^{+}$ or K${}^{+}$, as these are the most abundant monovalent cations in cells. Potassium ions are preferred over sodium ions, because the dehydration prior to their entry in the central channel of stacks formed by G-tetrads (the GQ core) is energetically less favourable for Na${}^{+}$ than K${}^{+}$. In contrast to Mg${}^{2+}$, which has a greater affinity for negatively charged backbone phosphates, monovalent cations (e.g., Na${}^{+}$ and K${}^{+}$) are the most commonly associated with non-anionic oxygens (i.e., oxygen atoms other than those in the phosphate group) as inner shell ligands. This general trend has been confirmed by Balasubramanian and co-workers in their experiments with a hybrid RNA molecule, which was equally likely to fold into two mutually exclusive intramolecular structures: a helix-based hairpin and a G-quadruplex. The eleven-nucleotide RNA-stretch (rGCGGGAGUGGG) experienced structural ambivalence between the hairpin and G-quadruplex conformers, as it is complementary to both (CUACCCGC) and (AGGGAGGG) sequences within the same strand [15]. The authors suggested that the conformational preference in that RNA molecule depended strongly on the relative amounts of mono- (K${}^{+}$) and divalent (Mg${}^{2+}$) metal cations, leading to quadruplex or hairpin conformations, respectively. The space between the four carbonyl groups is sufficiently large to accommodate coordinated sodium ions in the planes of G-tetrads. The ionic radius of Na${}^{+}$ is 0.95 Å, and experimental studies indicate that this is around the maximum size of a cation that can be coordinated in the plane of a G-tetrad (Figure 2).

Larger cations, such as K${}^{+}$ (with the ionic radius of 1.33 Å), are too large to be coordinated within the plane of the G-tetrad, but they are coordinated between the planes of two stacked tetrads. Multiple cation coordination geometries and occupancies are also possible and observed in G-quadruplexes [5]. The coordinated cations not only screen electrostatic repulsion between the lone pair electrons on carbonyl oxygens, but they also repel one another within the GQ core, when it contains more than one coordinated cation. Thus, the cation localization within a G-quadruplex is the result of both cation–lone pair attraction and cation–cation repulsion forces. In this work, we present an additional role of the cation in the folding and stabilization of G-quadruplexes, in the initiation of intramolecular charge transfer (valence tautomerism) in guanines, prior to the assembly of the adjacent guanines by hydrogen bonds via Hoogsteen edges. Thanks to the presence of a 2${}^{\prime }$hydroxyl group on ribose sugar, an RNA G-quadruplex forms a more thermodynamically stable, compact and less hydrated structure than its DNA counterpart [16,17,18,19]. The inherent chemistry of 2${}^{\prime }$hydroxyl groups on riboses plays a significant role in redefining the hydration structure in the grooves and the hydrogen bonding network, causing more intramolecular interactions within the RNA quadruplex topology and leading to enhanced stability. Furthermore, the 2${}^{\prime }$hydroxyl groups exert conformational constraints on the GQ architecture, preventing it from attaining syn-conformation (prerequisite for the antiparallel relative orientation of the G-strands), which limits the RNA quadruplexes to parallel conformations only. In the parallel conformation, the G-strands are oriented in the same direction and (preferably short) propeller loops connect the top of one strand with the bottom of the adjacent strand. Moreover, the presence of planar uridines instead of thymines (possessing a bulky methyl group) in the loops additionally stabilizes RNA quadruplexes, by promoting pi-stacking interactions with other bases and the release of the structured water molecules [20]. Overall, the monomorphic all-parallel and all-anti topology substantially reduces the diversity of RNA G-quadruplexes (as compared to their DNA counterparts [21]), making them very straightforward testing macromolecules for studying quadruplex folding and probing their structural modifications.

To understand how optimized RNA sequences could assist in achieving G-quadruplex topologies with desired physicochemical properties, we examined the effect of the loop sequences and cap compositions on the thermal stability and folding topology in a library of a dozen two-tetrad RNA G-quadruplexes by a combination of quantum mechanical (QM) calculations and molecular dynamics (MD) simulations, using classical and (for selected GQ systems) polarizable force fields [22,23]. The choice of the two-tetrad RNA G-quadruplexes as the testing system was dictated by their intrinsic lower stability due to minimal number of G-tetrads, causing higher sensitivity to structural modifications. For this reason, the influence of selective in silico mutations, introduced in the loop and cap fragments, on the dynamic behaviour and thermal stability of the investigated GQ topologies could be observed within ∼100 nanoseconds of MD simulations [24,25,26,27,28,29].

## 2. Materials and Methods

### 2.1. Quantum Mechanical Calculations

The molecule builder–editor, implemented in the Avogadro 1.2.0 [30] software, was used for the creation of the set of investigated guanine systems (G-monomer, two G-dimers, G-trimer and G-tetramer), as well as for the analysis of their molecular and electronic structures obtained from the QM calculations. The optimization of guanine systems, recommended for studying quadruplexes, was completed at the B3LYP-D3 level of theory, i.e., with the dispersion correction added explicitly by Grimme’s method (third order) with Becke–Johnson damping [31] and the 6-311++G(d,p) basis set [32], implemented in the Gaussian 16 package [33]. The semiempirical dispersion correction scheme that we used in this study provides reliable insights into the molecular structure and non-bonded interactions in assemblies of heterocyclic systems (in this case assemblies of nucleotides), which are in good agreement with the results that we later obtained with the LC-DFT approach, recently recommended for studies of GQ models [34,35].

### 2.2. Molecular Dynamics Simulations

The stability benchmark of the investigated ∼40 GQ topologies was conducted with MD >100 ns simulations using the Amber ff99SB-ILDN [36] force field (with the ParmBSC0 [37] nucleic acid parameters included) with explicit solvation (TIP4P [38] model) implemented in the GROMACS 4.5.x [39,40] package. The K${}^{+}$ ions were added only to neuralize the net charge in the simulated systems. Such depletion of ions with respect to the physiological conditions was dictated by the need to increase the sensitivity of the studied quadruplexes to structural modifications when cations leave the GQ core. To retain the GQ topologies of the models, the relaxation of these structures was performed gradually. First, only the positions of water molecules were optimized; then, only the backbone (P atoms) was relaxed during the NVT simulations. Finally, the whole GQ structure was involved by the NVT and then NpT simulations. After such optimization with the gradual release of a quadruplex structure, long MD production runs at room temperature (300 K) were performed. The Berendsen thermostat and Parrinello–Rahman barostat were used, where appropriate, in periodic boundary conditions.

The preparation of selected GQ systems for MD simulations with a polarizable force field included explicit solvation with TIP4P water and ions (Na${}^{+}$ and CL${}^{-}$ in 150 mM concentration) with GROMACS 2022.1 (Amber ff99sb-ILDN force field). The choice of salt concentration was dictated by buffer compositions used in experimental studies on RNA, mimicking physiological conditions, as well as reported recommendations for solvation in MD simulations of biomolecular systems [28,41,42,43]. The gradual minimization involved consecutive steps: minimization of the solvent with frozen nucleic acids and flexible water, minimization with position restraints on the nucleic acid with flexible water, unrestrained minimization with flexible water, and, finally minimization with rigid water molecules. All of the above steps consisted of the steepest descent followed by conjugate gradient phases. Next, the systems were converted to Tinker format with Tinker 8.10.2 [44] and an AMOEBA 2018 [45] polarizable force field, using an in-house script. Then, the systems were minimized with Tinker9 1.0.0 [46] and gradually heated to 300 K under NVT conditions. After NVT and NpT equilibration runs (1 ns long with a 1 ps time step) production runs of 60 to 103 ns were performed with Tinker9. A Nosé–Hoover thermostat and barostat were used, where appropriate, in periodic boundary conditions.

The trajectories from all MD simulations were visually examined and analysed using VMD 1.9.3 software [47]. The binding pocket detection was performed with GHECOM software [48]

### 2.3. De Novo Modelling of G-Quadruplex Topologies

We have developed a new software program, NAB-GQ-builder, based on Nucleic Acid Builder (NAB), which allows for the rapid, sequence-based building of initial quadruplex models, which can be further studied with any simulation/analysis methods [49]. NAB is a molecular manipulation language which is distributed within AmberTools and Amber molecular simulation packages [50]. As the main input, the program takes a sequence of the quadruplex to be built, and information on the expected interactions between guanine residues that create the quadruplex core. The latter consists of all pairs of guanines that should form hydrogen bonds via Hoogsteen edges, as well as pairs of guanines that are required to interact via pi-stacking. Based on these input data, the structure of the quadruplex is built by “growing” the programmed topology iteratively, i.e., one residue at a time. After each elongation of the nucleic acid strand, the model undergoes a series of structural transformations that allow for putting the obtained nucleotides in their desired geometric configuration. The whole procedure can be outlined as the following steps:

1. Adding a new nucleotide to the model. The orientation of the new nucleotide with respect to the already built ones (if any) resembles that in canonical helical nucleic acids—with adjacent nucleotides interacting via pi-stacking.
2. Application of the geometric restraints to the model based on user’s input and a predefined set of templates, consisting of pairs of nucleotides involved in hydrogen bonds via Hoogsteen edges, and pairs of pi-stacking nucleotides.
3. Concerted pulling of residues, based on the imposed restraints. The conjugated gradient (CG) algorithm is used for this purpose, as implemented in NAB for “rigid body transformations”. The structure with the lowest potential energy is chosen as the input configuration for the next step.
4. Capping and parametrization. The model is properly capped to represent a chemically valid nucleic acid molecule with Amber ff99bsc0-OL3 [37,51,52] force field parameters assigned. assigned.
5. The backbone with restrained nucleobases is optimized using the Generalized Born implicit solvent model [53].
6. The optional removal of the cap prior to the addition of a new nucleotide.

If the quadruplex-building procedure goes wrong at any point (i.e., the geometric constraints cannot be fulfilled due to entanglement), it can be easily detected by a sudden increase in potential energy signalled at step 3. This problem can be dealt with by a manual intervention (i.e., placing the problematic nucleotide in a proper orientation and restarting the program). In the case of all quadruplexes built for this study, it was enough to increase the number of CG trials in step 3 to solve this problem automatically. The output quadruplexes lack the central ions, and have to be added manually. The final GQ model shall undergo standard energy minimization in explicit solvent before further investigations. Further development of the program is planned. The current version of the NAB-GQ-builder is available upon request. An exemplary building process performed by the program is presented in Appendix C.

## 3. Results

**Figure 3:**
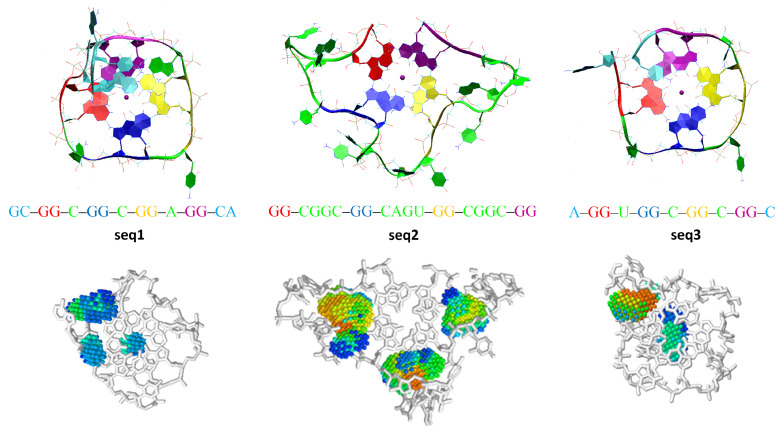
**Top**: The three RNA G-quadruplex systems located in the 5${}^{\prime }$-UTR of the *KRAS* transcript, which are stable at room temperature according to molecular dynamics simulations with a polarizable force field (AMOEBA). Seq1 and seq3 are both very stable thanks to their single-nucleotide loops and cap fragments. **Bottom**: Assessment of the binding pockets in the seq1, seq2 and seq3 GQ topologies. The deep potential binding pockets are located inside long loops of the least stable seq2 quadruplex, which could be stabilized with small molecules.

In the first step of our study, we probed the effect of the single-stranded sections (i.e., loops and caps) on the thermal stability and folding topology in a library of ∼40 designed GQ systems, and three RNA quadruplexes located in the 5${}^{\prime }$-untranslated region (5${}^{\prime }$-UTR) of the *KRAS* transcript (Table A1 in Appendix B.1) [54]. To this end, we built all of the investigated systems, using our new tool for de novo quadruplex folding (NAB-GQ-builder, see Materials and Methods, Section 2.3), and performed MD runs on a series of GQ models (Table A1, Appendix B.1). Each investigated sequence could be described as leftCap–GG–leftLoop–GG–centralLoop–GG–rightLoop–GG–rightCap. The reference sequence (PDB ID: 2RQJ, Figure 1, Table A1, Models 1 and 2) of the investigated series of RNA quadruplexes was GG–A–GG–A–GG–A–GG–A, i.e., had single-nucleotide adenine loops and one single-nucleotide adenine cap [55]. Except in the case of G-undecamer (Table A1, Model 24), where guanines from the loops caused a structural destabilization by strongly interfering with G-tetrads, all investigated GQ topologies with single-nucleotide loops and no cap fragments remained stable (Table A1, Models 16, 24–26), in agreement with previous reports [12,55]. The presence of at least one single-nucleotide cap substantially enhanced the stability of all those GQ structures (e.g., even a G-undecamer quadruplex with one A-cap remained stable, Table A1, Model 5), primarily through retaining the central cation inside the GQ core, but also by contributing to the stabilizing pi-stacking interactions with the G-tetrad residues (Table A1, Models 2–6). The two investigated *KRAS* quadruplexes, GC–GG–C–GG–C–GG–A–GG–CA (Figure 3, seq1, Table A1, Model 39) and A–GG–U–GG–C–GG–C–GG–C (Figure 3, seq3, Table A1, Model 41), fell into the category of highly stable GQ topologies with single-nucleotide loops and two cap fragments.

Interestingly, regardless of the composition of other mutated single-stranded GQ fragments, a single uridine in the central loop always guaranteed the highest stability of a G-quadruplex structure (Table A1, Models 4, 6, 26, 31). As anticipated, single-uridine loops promoted the stabilization of GQ structures by interacting with structured water molecules and by possessing efficient hydrogen bond acceptors (i.e., carbonyl oxygens). Moreover, by being planar, and the smallest and the most symmetric of the nucleotides, two uridines in the propeller loop promoted its stiffness by effective pi-stacking interaction, which was unattainable for any other two-nucleotide loop. Still, the investigated G-quadruplexes with two-uridine loops were stable only if ended by two-nucleotide caps on both sides (i.e., single-nucleotide caps were not enough), with decreasing stability following this trend: GC–, CA–, AU–, AC–, GU– (Table A1, Models 19, 22, 20, 18, 38, respectively). As mentioned earlier, cap fragments can stabilize a quadruplex by preventing the escape of cations from the GQ core, and to some extent by fostering pi-stacking interactions with the nearest G-tetrad. Importantly, the latter could not be achieved when cap nucleotides interacted too strongly with G-tetrad or loop residues, and caused reciprocal displacements of the guanines within the tetrad, leading to a gradual dissociation of the quadruplex.

Overall, the interactions between caps and G-tetrads could be moderated, for example, by preferentially separating pi-stacking with the core guanine loop residues (i.e., purines) by pyrimidine linkers. The optimization of the interactions between the caps and loops was much more challenging. A successful interplay of interactions between purine–pyrimidine caps and two-uridine loops that stabilized G-quadruplexes were observed for the GC–, AC–, and AU– caps (but not GU–, where both cap residues possess carbonyl oxygens, which are highly reactive with cations and water molecules). Interestingly, one loop with the opposite order of purine and pyrimidine residues (CA–) also promoted the stabilization of a GQ system with two-uridine loops (Table A1, Model 18). Thanks to a relative chemical neutrality (i.e., possessing the smallest dipole moment among nucleotides and no carbonyl oxygens), adenine worked successfully as a linker residue of that cap. The stabilizing effect of adenine-containing caps was also observed in NMR experiments on a G-quadruplex-forming human *c-MYC* oncogene promoter [56]. Although the three-nucleotide loops were generally found to be too labile to foster the stability of G-quadruplexes, some combinations of nucleotides in those loops resulted in relatively stable topologies (Table A1, Models 9, 13–15, 27–29). In the case of a purine–purine–pyrimidine loop (Table A1, Model 15), two purines stiffened the loop, preferably by pi-stacking with each other, and in this way promoted the stability of a GQ system, in agreement with previously reported experimental studies on quadruplex loops [7]. In the case of a pyrimidine–purine–pyrimidine loop (Table A1, Model 13), by separating a purine residue from G-tetrads by a pyrimidine linker, the core guanines were protected from potentially disturbing stacking interactions with the purine loop residue, and in this way maintained the integrity and stability of such a quadruplex. We found a similar trend in the third studied *KRAS* quadruplex, GG–CGGC–GG–CAGU–GG–CGGC–GG (Figure 3, seq2, Table A1, Model 40), where in each loop the two preferentially pi-stacking purine residues in the middle were separated from G-tetrads by pyrimidine linkers. The dynamic behaviour of the seq2, the least stable among *KRAS* quadruplexes, drew our attention to the influence of the central loop on the overall stability of GQ structures. As investigated in detail and described in the Appendix B.2, an escape of the central cation from the GQ core of the seq2 quadruplex and a collapse of the central loop both led to a pronounced distortion of the guanine core prior to quadruplex unfolding. We also observed that even a minor intervention in the central loop’s content (e.g., adding/changing nucleotides) very strongly influenced the stability of the whole G-quadruplex (Table A1, Models 13 and 14). These results are consistent with experimental assessment of the influence of the GQ loop sequence (especially the central loop) on the quadruplex topology and stability [57,58].

**Figure 4:**
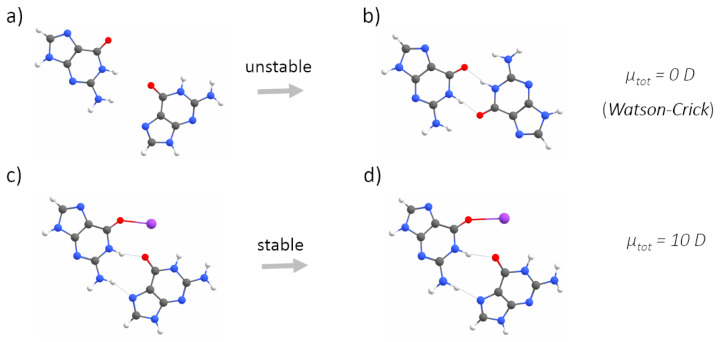
The optimizations of two adjacent guanines without (**a**→**b**) and with (**c**→**d**) metal (here potassium) cations, calculated at the QM level of theory. The optimization without a cation leads to the cancellation of the dipole moments by a pronounced mutual rearrangement of the guanines, driving the residues to interact with hydrogen bonds via Watson–Crick edges. The presence of the cation stabilized the mutual orientation of guanines, favouring hydrogen bonding via Hoogsteen edges, despite a non-zero total dipole moment of the system.

**Figure 5:**
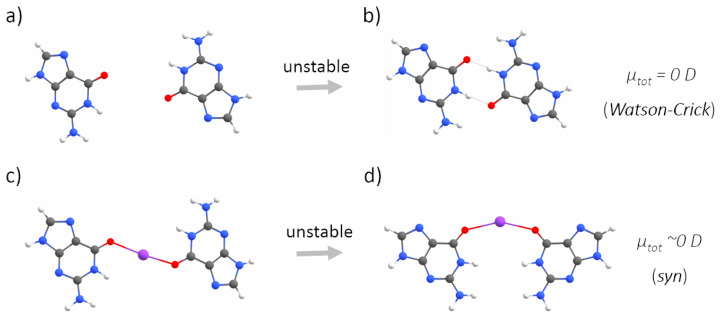
The optimizations of two diagonally opposed guanines without (**a**→**b**) and with (**c**→**d**) a metal (here, potassium) cation, calculated at the QM level of theory. The optimizations in both cases were associated with the cancellation of the dipole moments and pronounced changes in the mutual orientation of guanines, that (in both cases) preclude the formation of GQ topology.

The second step of our study concerned better understanding the role of electrostatic interactions in G-quadruplex folding and stabilization. To this end, we built five simplified (i.e., one layer of guanines, and no backbone) models, representing all possible stages of a GQ-forming process: a G-monomer, two G-dimers, a G-trimer and a G-tetramer in the reciprocal configurations characteristic of a G-tetrad. This simplified model of a G-quadruplex was justified by the similarities in the values and directions of the dipole moments calculated for a single guanine and stacks of three guanines (Appendix A, Figure A2). We considered two cases of a G-dimer, with adjacent (Figure 4a) or diagonally opposed guanines (Figure 5a). Adding the adjacent guanine to a G-monomer ($\mu_{tot}$ = ∼7 D), initially resulted in an increase in the total dipole moment ($\mu_{tot}$ = ∼10 D).

**Figure 6:**
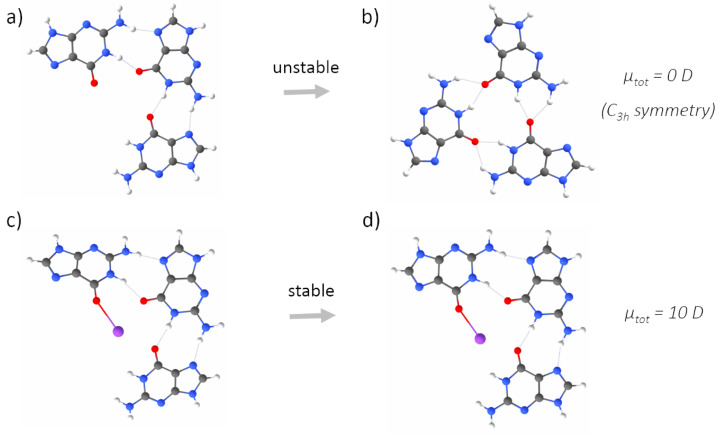
The optimizations of three adjacent guanines without (**a**→**b**) and with (**c**→**d**) a metal (here, potassium) cation, calculated at the QM level of theory. The optimization without a cation leads to the cancellation of the dipole moments by a reorganization of the guanines, leading to a triangular orientation stabilized by Hoogsteen-like hydrogen bonds. The presence of the cation stabilized the mutual orientation of guanines characteristic of a G-tetrad, via the Hoogsteen fingerprint of hydrogen bonds, despite a non-zero total dipole moment of the system.

**Figure 7:**
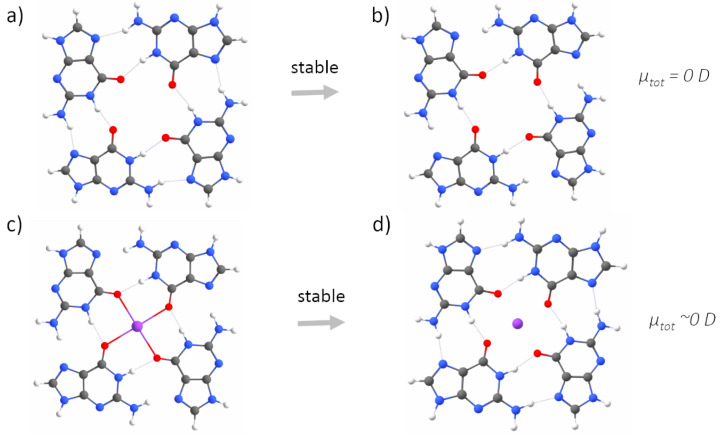
The optimization of a G-tetrad without (**a**→**b**) and with (**c**→**d**) a metal (here, potassium) cation, calculated at the QM level of theory. The optimization in both cases did not much change the mutual arrangement of the guanines. The potassium ion moved out of the plane of the G-tetrad upon optimization. A G-tetrad without a cation remains stable, as guanines are held together by the attractive forces originating from the cancellation of dipole moments, along with the circular network of hydrogen bonds via Hoogsteen edges.

**Figure A1:**
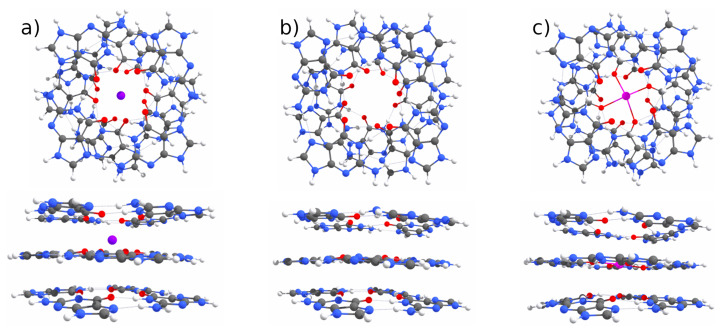
The structures of three layers of stacking guanine quartets, optimized at the QM level of theory, shown as simplified (i.e., lacking single-stranded motifs) representations of G-quadruplexes with coordinated K${}^{+}$ (**a**), Na${}^{+}$ (**c**), and without cations (**b**). Note that without flexible loops, the quartets remain intact even without cation coordination, thanks to the cancellation of the dipole moments within the G-tetrads combined with the circular network of the hydrogen bonds via Hoogsteen edges. The adjacent G-tetrads are held together by the quadrupole interactions between pi-stacking guanines.

**Figure A2:**
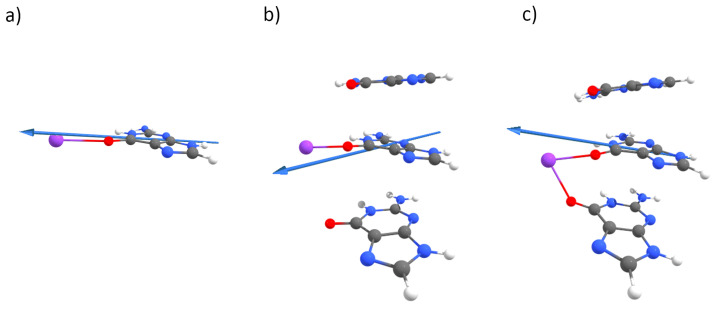
The dipole moments calculated at the QM level of theory for a single guanine coordinating K${}^{+}$ (**a**), a three-guanine stack with K${}^{+}$ situated in the plane of the middle guanine (**b**), and a three-guanine stack with K${}^{+}$ situated between the planes of two guanines (**c**). Note the similarity in the values (i.e., the length of the vector) and directions of the dipole moments obtained for all three cases.

Then, upon optimization, the residues sampled the conformational space until they were connected by two N–H⋯O hydrogen bonds via Watson–Crick edges, which was associated with the cancellation of the dipole moments ($\mu_{tot}$ = ∼0 D, Figure 4b). The cancellation of the dipole moments was also observed among two diagonally opposed guanines (Figure 5b). Again, the dipole–dipole attractive force led the guanines to the same final mutual orientation as for the adjacent guanines, followed by the formation of two N–H⋯O hydrogen bonds via Watson-Crick edges. From this part of the study, we conclude that the hydrogen bonding via Hoogsteen edges, which is characteristic of G-quadruplexes, is in general unfavourable for two interacting guanine residues (Figure 4b and Figure 5b). Instead of forming hydrogen bonds via Hoogsteen edges, during optimization, the neighbouring guanines always (i.e., even when additionally separated by 5 Å) sampled the conformational space until they could finally connect by favourable hydrogen bonds via Watson–Crick edges and cancel the dipole moments. The model of the G-trimer, in order to cancel its substantial total dipole moment ($\mu_{tot}$ = ∼13 D, Figure 6a), became triangular during optimization (Figure 6b), while both the initial (Figure 7a) and optimized (Figure 7b) models of a complete G-tetrad resulted in a zero total dipole moment. The result presented in Figure 6b is in agreement with the two-decade-old studies of S̆poner and co-workers on G-trimer intermediates, showing that without the cation “well-structured parallel-stranded all-anti triplexes are unlikely to participate in folding/formation process of G-DNA” [59,60]. As in the other studied assemblies, the cancellation of the dipole moments corresponded to attractive forces, which in the case of the G-tetrad worked along with the circular network of hydrogen bonds via Hoogsteen edges (Figure 7b). The introduction of a monovalent (Li${}^{+}$, Na${}^{+}$ and K${}^{+}$) metal ion to the same five guanine systems confirmed the game-changing role of cations in the formation of G-quadruplexes. For example, upon interaction with a potassium cation, the dipole moment of the G-monomer increased (from ∼7 to ∼10 D). The relatively high value of the total dipole moment did not change when another adjacent (Figure 4c) or diagonally opposed (Figure 5c) guanine was added to that G-monomer. Interestingly, the interactions with a cation stabilized only the adjacent guanines in the Hoogsteen hydrogen-bonded orientation (Figure 4d), while in the case of the diagonally opposed G-dimer, one guanine rotated by 180${}^{\circ }$ during optimization (Figure 5d). Adding the third guanine to the system still did not change the total dipole moment ($\mu_{tot}$ = ∼10 D, Figure 6c), while the cation preserved the initial Hoogsteen hydrogen-bonded arrangement of guanines during optimization (Figure 6d). These results are in agreement with several previous MD studies on the folding pathways of G-quadruplex structures [59,61], as well as NMR experiments on G-triads coordinating cations [62]. Finally, the system of four guanines (Figure 7c) exhibited an excellent stability, associated with the zero total dipole moment (Figure 7d) and the circular network of hydrogen bonds via Hoogsteen edges. As expected, the too-large potassium cation moved out of the G-tetrad’s plane during optimization, while smaller cations (Li${}^{+}$ and Na${}^{+}$) stayed inside the negatively charged GQ core (Figure 2). The extended models of G-strands, represented by stacks of three guanines, exhibited analogical trends in forming guanine assemblies without and with the assistance of cations. Interestingly, by coordinating only one metal ion, the whole isolated stack of three guanine tetrads kept the proper geometry during optimization, as the stabilization effect was propagated on the neighbouring guanines in the stack by a combination of pi-stacking and dipole–dipole interactions (Figure A1 and Figure A2 in Appendix A). The high charge density of Li${}^{+}$ (0.90 Å) was responsible for the disrupted planarity of the tetrad formed by crowded (i.e., too strongly attracted by the too small cation) guanines, and corresponded to the lowest stabilization effect. In contrast, one K${}^{+}$ ion effectively coordinated eight guanines from the two neighbouring G-tetrads and all pi-stacked layers maintained their planarity. The observed trend in the coordination strength (i.e., K${}^{+}$ > Na${}^{+}$ > Li${}^{+}$) is particularly pronounced in guanine-deficient GQ systems, which, by virtue of possessing the minimal number (two) of pi-stacking G-tetrads, depend even more strongly on the stabilization effect provided by the coordination of cations (Table A1 in Appendix B.1).

## 4. Discussion

**Figure 8:**
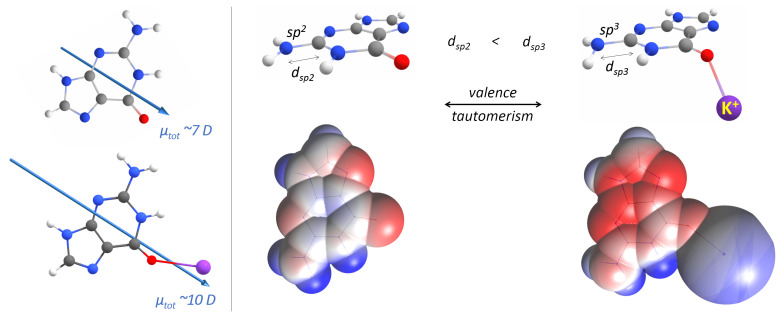
**Left**: The dipole moments calculated for guanine alone (**top**) and upon coordination of a metal cation (**bottom**) at the QM level of theory. **Right**: The valence tautomerism observed in guanine upon the coordination of a metal (here, potassium) cation. The intramolecular charge transfer that corresponds to the sp${}^{2}$ to sp${}^{3}$ transition of the amino group (**top**). The length of the C–N bond in the amino group was calculated as 1.362 and 1.371 Å for sp${}^{2}$ and sp${}^{3}$ hybridizations, respectively. The electrostatic potentials mapped on the van der Waals surfaces (**bottom**). Red and blue colours correspond to negative and positive charges, respectively. Note the increase in the negative charge (more red region) on the nitrogen in the amino group upon coordination of the cation, which corresponds to the sp${}^{3}$ hybridization.

Our quantum chemical calculations on the above-described guanine systems strongly indicate the key role of the cation in the process of G-quadruplex folding. Curiously, we observed the electronic effects of metal ion coordination on the intramolecular charge transfer (valence tautomerism) in guanines (Figure 8), manifested by the conformational transformation (planar→nonplanar) of the amino group (–NH${}_{2}$), associated with the change in its hybridization (sp${}^{2}$→sp${}^{3}$). The phenomenon of tautomerism has already been observed in RNA biochemistry and proposed, for example, to enhance the structural and functional diversity of RNA enzymes and aptamers [63,64]. To the best knowledge of the authors, valence tautomerism has not yet been reported for RNA systems. However, it was reported for 4-aminocoumarin, which, similarly to guanine, possesses the amino group attached to a heterocyclic ring and undergoes transitions between sp${}^{2}$ and sp${}^{3}$ hybridization states [65,66]. An isolated guanine possesses an sp${}^{2}$ hybridized (i.e., planar) amino group with the nitrogen’s lone electron pair delocalized into rings (Figure 8). We observed that the coordination of a metal ion by guanine promoted sp${}^{3}$ hybridization, associated with the flattened triangular pyramidal geometry of the –NH${}_{2}$ group (i.e., the donor in the hydrogen bond via Hoogsteen edges). The sp${}^{3}$ hybridized amino group, being more flexible and stretched than its sp${}^{2}$ counterpart, becomes more easily reachable by the hydrogen bond acceptor on the adjacent guanine. Interestingly, once the sp${}^{3}$ hybridized amino group creates the hydrogen bond with the acceptor, the planarity of the –NH${}_{2}$ group is restored, which enhances the ability of that amino group to donate hydrogen bonds [67].

Based on these observations, we postulate that the valence tautomerism is an important element in the cation-initiated G-quadruplex folding mechanism.

**Figure 9:**
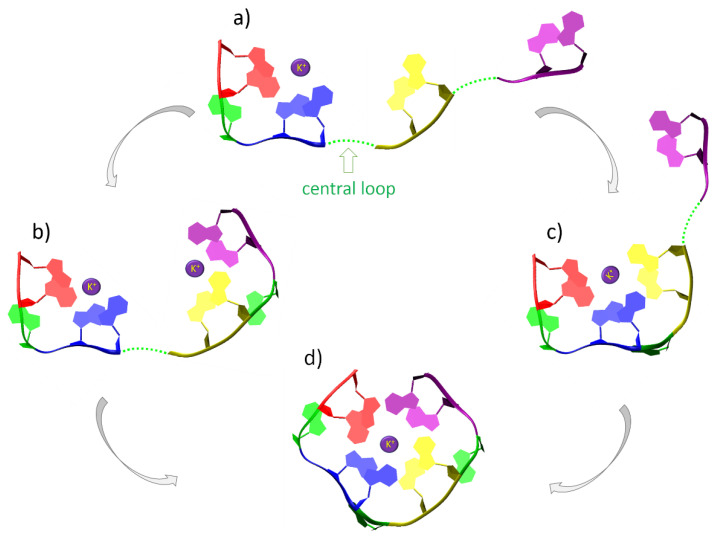
Two possible scenarios of G-quadruplex folding proposed on the basis of the obtained QM results. Grey arrows indicate the sequences of folding, while green dotted lines indicate unfolded loops. The “hinge scenario” (**a**→**b**→**d**): First, two adjacent pi-stacked G-strands assemble upon interaction with a metal cation (**a**), and the same happens to the adjacent pair of G-strands (**b**); then, due to a hinge-like conformational transformation of the central loop, both GQ halves assemble into a complete quadruplex topology (**d**). Note that the “hinge scenario”, would require an energetically unfavourable dissociation of an extra cation coordinated to one dimer of the adjacent G-strands before completion of the folding process. Otherwise, the two halves of a quadruplex could not assemble due to repulsion forces between the two cations. The “sequential scenario” (**a**→**c**→**d**): After two adjacent pi-stacked G-strands assemble upon interaction with a metal cation (**a**), the next G-strands join them in a sequential manner, i.e., through the stage of three adjacent G-strands (**c**), until they fold into a complete quadruplex (**d**). Note that the “sequential scenario” requires a high propensity of the central loop for folding into a propeller single-stranded structure in order to assemble the third G-strand.

Without the assistance of a cation, neither two adjacent guanines extracted from a G-tetrad, nor stacks of guanines (representing G-strands), could preserve the reciprocal orientation that allows for hydrogen bonding via Hoogsteen edges. Instead, due to the dipole–dipole attractive forces between guanines, they are conformationally rearranged during optimization until they finally interact by hydrogen bonds via Watson–Crick edges and cancel the total dipole moment (Figure 4b and Figure 5b) [68,69,70]. We demonstrated that the coordination of a cation is crucial for the stabilization of all studied G-tetrad fragments (Figure 4c→d, Figure 6c→d, Figure 7c→d), except for the case of the diagonally opposed G-dimer (Figure 5c→d). In that case, the coordinated cation promoted a 180${}^{\circ }$ rotation of one guanine, corresponding to its relative syn-conformation, which is prerequisite for the antiparallel relative orientation of the G-strands, and is therefore unavailable for the intrinsically parallel topology of RNA G-quadruplexes. Based on our conclusions from the analysis of the simplified model of tetrad-forming guanine systems (Figure 3, Figure 4, Figure 5, Figure 6 and Figure 7), we propose a folding mechanism of the monomorphic (i.e., all-parallel stems with all-anti guanine orientation) intramolecular RNA G-quadruplexes, presented schematically in Figure 9 as the a→c→d scenario. When the adjacent G-strands fold sequentially around the cation (i.e., the “sequential scenario”), the total dipole moment of the assembly stays the same throughout the whole process (as in Figure 4d and Figure 6d). The electrostatic attraction between carbonyl oxygens and a coordinated cation compensates for the non-zero (but constant) total dipole moment of the RNA system during the intermediate stages of folding, until the full G-quadruplex is created and the dipole moments on guanines are finally cancelled (as in Figure 7d).

Importantly, without the assistance of a metal cation, the guanines from adjacent G-strands are unable to attain the mutual configuration that is prerequisite for hydrogen bonding via Hoogsteen edges (Figure 4). Therefore, we propose the “sequential scenario” as the mechanism of G-quadruplex folding, which requires cation coordination to initiate and foster the quadruplex-forming process.

The proposed mechanism of sequential G-quadruplex folding (Figure 9, scenario a→c→d) provides the answer to the question “why do tetrameric quadruplexes experimentally tend to form all-parallel stems with all-anti nucleobase orientation” [34].

**Figure A4:**
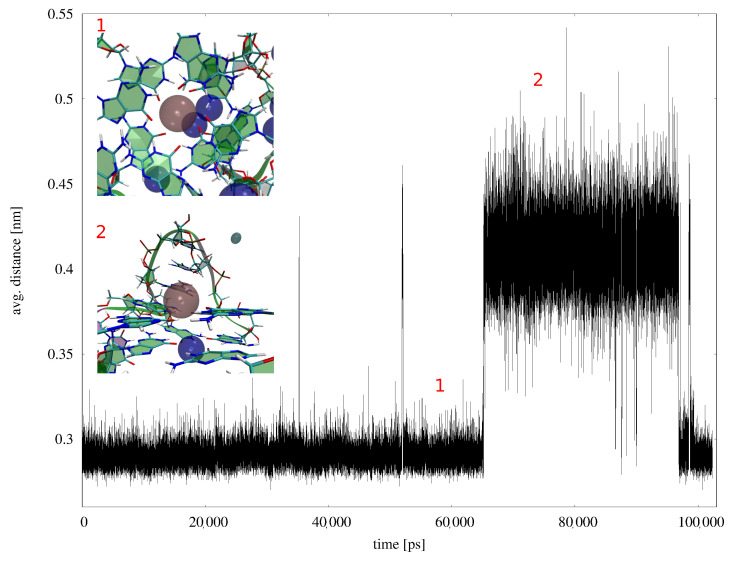
The stability of the seq2 quadruplex, as expressed by the average distance between K${}^{+}$ and the carbonyl oxygens of the eight guanines coordinating it. Inlet 1—the stable GQ core structure. Inlet 2—K${}^{+}$ (dark-pink sphere) left the GQ core due to the repulsion of Na${}^{+}$ (dark-blue sphere) at the entrance of the GQ core, and due to the deformation of the GQ core (see the text of Appendix B.2 and Figure A6 for details).

**Figure A6:**
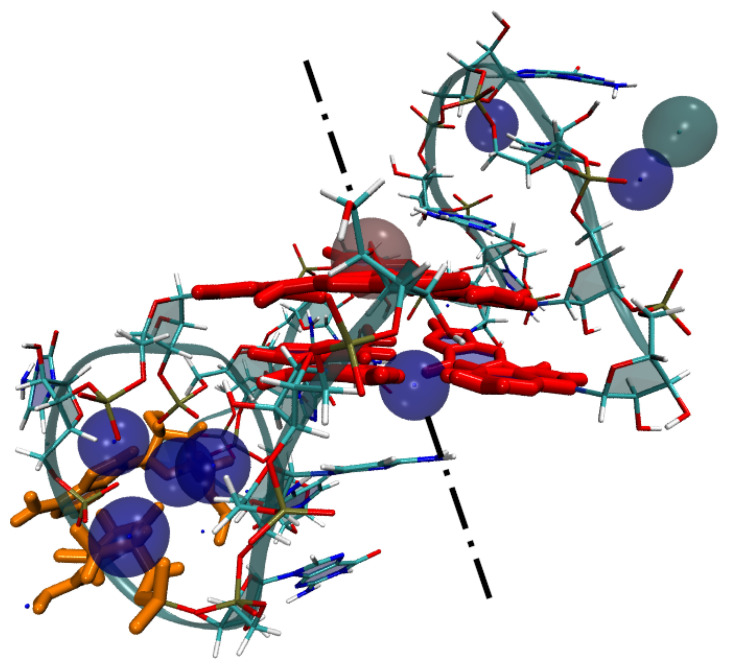
One of the most significant deformations observed for the seq2 quadruplex. The central loop surrounds four Na${}^{+}$ ions (dark-blue spheres, down left), which—enhanced by inter-loop pi-stacking (orange)—tightens the structure in this region. The resulting sheer force acts on the core guanines (red), leading to the deformation of the GQ core. The dashed line shows the skewed main axis of the GQ core.

The stability of the complete quadruplex structure, which depends on the cancellation of the dipole moments and the circular network of hydrogen bonds via Hoogsteen edges (Figure 7b) within G-tetrads, is additionally moderated by the dynamic behaviour of the single-stranded fragments, with the key influence of the central loop on the stabilization of the whole GQ topology. The latter effect is strongly pronounced in the proposed “sequential scenario” of RNA G-quadruplex folding, where the propensity of the central loop for folding into a propeller single-stranded structure appears crucial for the creation of the intermediate with three assembled G-strands (Figure 9c). A stable G-quadruplex topology allows an exchange of ions between the negatively charged GQ core and the surrounding solvent (Figure A4–Figure A6). The most stable quadruplexes should remain intact even when cations completely leave the GQ core for a short time (Figure 7b). A longer absence of cations in the GQ core destabilizes the structure of a G-quadruplex, finally leading to its dissociation (see Table A1, GQs marked as “destroyed”).

We observed that the central loop had the biggest impact on the G-quadruplex stability and the highest susceptibility to changes; hence, this single-stranded section shall be designed with particular caution.

As anticipated, single-nucleotide (particularly uridine) loops promoted G-quadruplex stabilization, with the highest stability detected for GQ topologies possessing the single-uridine central loop. We provided guidance in stabilizing GQ topologies possessing longer loops, for example, by stiffening the loop by the placement of nucleotides that preferably pi-stack with each other, or by separating the preferably pi-stacking purine residues from the G-tetrads and caps. The single-nucleotide caps stabilized the GQ topologies indirectly by locking cations inside the GQ core, and directly by contributing to the stabilizing pi-stacking interactions with the G-tetrad residues. We observed that the fluctuations of the loops, especially those that were longer, were often coupled with the sequence-dependent dynamic behaviours of the cap fragments. The composition of the caps corresponded to their bulkiness and flexibility, which were both responsible for their (de)stabilizing properties. The larger (i.e., purine) the nucleotide, the better the cap’s coverage of the entrance to the GQ core, which could prevent the escape of the central cation. The best stabilizing single-nucleotide caps were made of adenine, which possesses the smallest dipole moment among nucleotides and no carbonyl oxygens. Thus, they did not destabilize G-tetrads by competing for interactions with the core guanines or cations. Other single-nucleotide caps had weaker stabilizing effects, dictated by their chemical characteristics and therefore their propensity to interact with core guanines and cations. Another way of moderating the stabilization of G-quadruplexes by capping was achieved by two-nucleotide caps, especially by the right combination of purine and pyrimidine residues. By using a pyrimidine nucleoside as a linker that moves a bigger purine residue away from the nearest G-tetrad, we moderated the pi-stacking interactions between the cap and guanines, allowing for higher flexibility and therefore efficiency in retaining cations inside the GQ core. Other compositions of two-nucleotide or longer caps had generally destabilizing effects on the studied G-quadruplexes, especially in the topologies possessing long and labile loops with nucleotides that were prone to interactions. The role of the caps as quadruplex stabilizers is particularly pronounced in the guanine-deficient topologies, which by default possess a weaker set of stabilizing interactions. In such cases, the efficient maintenance of the cation inside the core is crucial for preserving the integrity of the otherwise floppy tridimensional structures of two-tetrad G-quadruplexes.

In summary, the diversity of supramolecular interactions inherent to G-quadruplexes, including hydrogen bonding, pi-stacking, charge transfer interactions, and metal ion coordination-driven self-assembly, possess an inspiring methodological strategy of fabricating GQ-based functional materials [71,72,73,74]. Advanced G-quadruplexes with balanced topologies and, in turn, programmable physicochemical properties have broad prospective applications as stimuli-responsive (i.e., sensitive to metal ion concentration) and reversible smart biomaterials.

Furthermore, we performed a pocket-based druggability assessment of three *KRAS* two-tetrad RNA G-quadruplexes, whose stability we had assessed by molecular dynamics simulations using both classical and polarizable force fields (Appendix B). Inside the central CAGU loop of the least stable seq2 KRAS GQ topology with four-nucleotide loops and no caps (Table A1, Model 40) we found a deep potential ligand-binding pocket (Figure 3, bottom panel). As the pocket is located inside the most vulnerable element for the stabilization of the whole GQ topology (i.e., the central loop), it should be prone to intervention with small-molecule ligands. Moreover, the sequential diversity of the residues involved in this pocket should correspond to a high degree of selectivity. In summary, the three investigated *KRAS* oncogene promoter G-quadruplexes have potential as therapeutic targets for selective structure-stabilizing small-molecule ligands. This emerging potential was recently reported experimentally [54,75]. The possibility of building reliable tridimensional atomistic GQ models with NAB-GQ-builder makes the structure-based design of drugs targeting quadruplexes possible.

## 5. Conclusions

Considering that GQ-forming sequences are overrepresented in the oncogene promoter regions of the human genome, while being underrepresented in structural databases, we developed a software—NAB-GQ-builder—that builds de novo atomistic GQ-models based on nucleotide sequences. These models may become the basis for drug design studies targeting G-quadruplexes [76], whose experimentally resolved structures are not yet available.

We demonstrated, for the first time, an additional role of metal cations in the folding and stabilization of G-quadruplexes, in the initiation of intramolecular charge transfer in guanines (valence tautomerism). This phenomenon shall be further explored in the context of guanine’s potentially enhanced chemical reactivity, which may lead to the RNA oxidative damage observed in disease states, especially for neurodegenerative disorders.

Our combined simulations at the QM and MD levels of theory confirmed the game-changing role of cation coordination in the initiation of the sequential folding of G-quadruplexes, otherwise dictated by the cancellation of the dipole moments on guanines. This mechanism explains previous experimental reports on the spontaneous tendency of tetrameric G-quadruplexes to form all-parallel stems with all-anti nucleobase orientation.

Finally, by probing the influence of the composition of loops and caps on the dynamic behaviour of guanine-deficient quadruplex topologies, we investigated their sequence-dependent susceptibility to stabilization. On the basis of these results, we provided guidance for the optimization of GQ-based morphologies for development of functional RNA-based biomaterials [77].

## Figures and Tables

**Figure A3:**
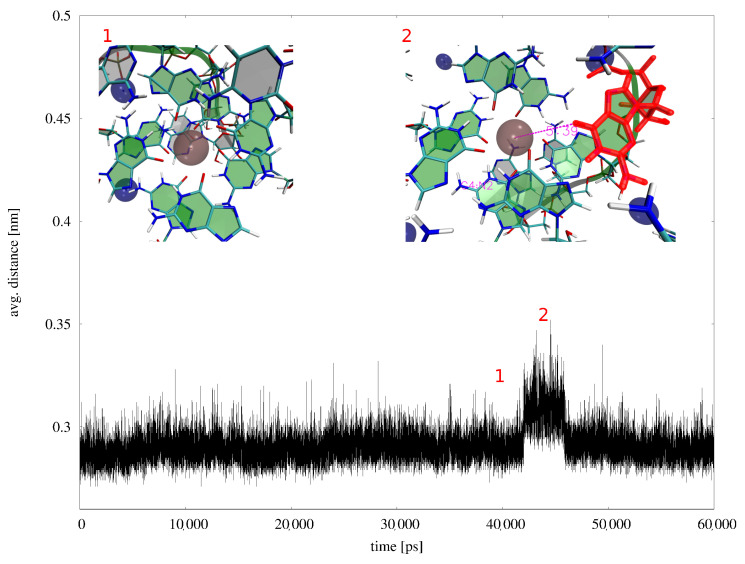
The stability assessment of the seq1 quadruplex, as expressed by the average distance between K${}^{+}$ and the carbonyl oxygens of eight guanines coordinating the ion. Inlet 1 (corresponding to the simulation time labelled on the chart at 40 ns of the simulation)—the stable GQ core structure. Inlet 2 (and label 2 on the chart)—the lateral guanine (red) diffused away from the central K${}^{+}$ (dark-pink sphere), which was reversed after ∼3 ns. See the text of Appendix B.2 for a detailed explanation.

**Figure A5:**
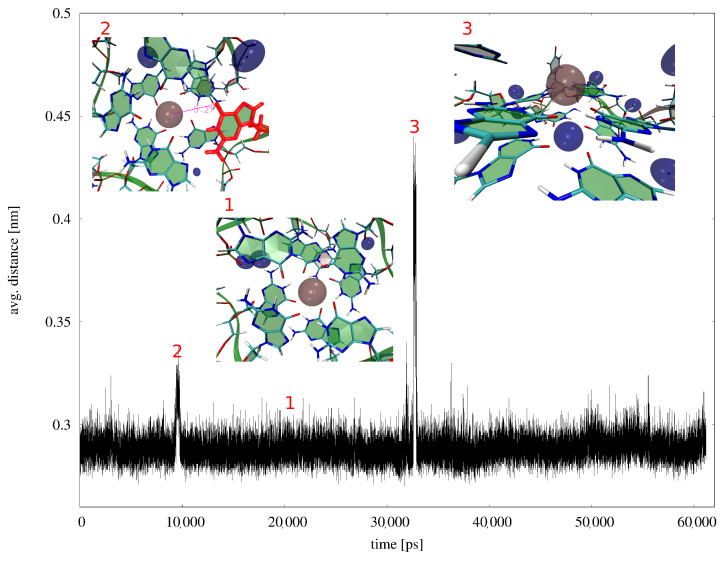
The stability of the seq3 quadruplex, as expressed with the average distance between the central K${}^{+}$ and the carbonyl oxygens of the eight coordinating guanines. Inlet 1—the stable GQ core structure. Inlet 2—one of the guanines (red) dissociated from the central K${}^{+}$ (dark-pink sphere) transiently, losing hydrogen bonding with the rest of the tetrad. Inlet 3—K${}^{+}$ was pushed out of the GQ core by Na${}^{+}$ (dark-blue sphere below K${}^{+}$) for ∼300 ps.

**Figure A7:**
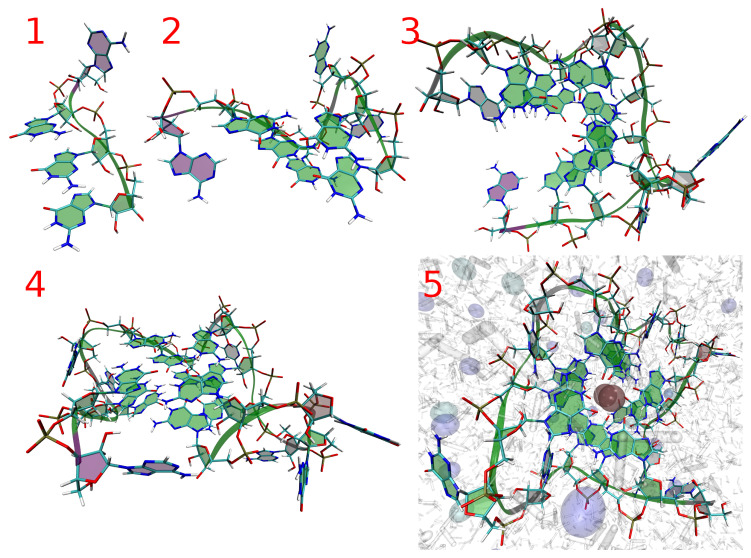
Stages of the folding simulation of an example G-quadruplex with the sequence A-GGG-AG-GGG-C-GGG-AGU-GGG-C, performed by means of the NAB-GQ-builder. (**1**) The first G-strand is ready. (**2**) One guanine is still missing in the second G-strand. (**3**) The third G-strand is ready. (**4**) Only one guanine is missing from the core. (**5**) The complete, slightly minimized quadruplex with manually added K${}^{+}$ ions (dark-pink spheres), in explicit water and ions.

## Data Availability

The data presented in this study are available on request from the corresponding author. The data are not publicly available due to significant size of the MD trajectory files. The NAB-GQ-builder still has not completed the documentation. Once the tutorial is ready, the software will be deposited in https://github.com/steady-blow-of-dirt/ repository (accessed on 15 September 2022).

## Abbreviations

The following abbreviations are used in this manuscript:

5${}^{\prime }$-UTR	5${}^{\prime }$-untranslated region
DFT	density functional theory
GQ	guanine quadruplex
MD	molecular dynamics
NAB	Nucleic Acid Builder
PDB	Protein Data Bank
QM	quantum mechanics
NMR	nuclear magnetic resonance

## Appendix B. MD Results

**Table A1:** RNA quadruplex-forming systems simulated with the Amber ff99-ILDN-Bsc0 force field. Items with dash (“-”) symbol in the sequence field represent dimerized quadruplexes.

Model	Type	Sequence	MD Time $\left[\mathrm{ns}\right]$	Result	Ion Escape $\left[\mathrm{ns}\right]$	Dissociation $\left[\mathrm{ns}\right]$
1	RNA	AGGAGGAGGAGG-GGAGGAGGAGGA	100	stable	-	-
2	RNA	GGAGGAGGAGGA	100	stable	-	-
3	RNA	GGAGGGGGAGGA	100	stable	-	-
4	RNA	GGAGGUGGAGGA	100	stable	-	-
5	RNA	GGGGGGGGGGGA	100	stable	-	-
6	RNA	GGUGGUGGUGGA	100	stable	-	-
7	RNA	GGAAGGAAGGAAGGA	100	destroyed	10	9.8
8	RNA	GGGGGGGGGGGGGGA	100	destroyed	63	49
9	RNA	GGAGGAGAGGAGGA	100	destroyed	26.1	26
10	RNA	GGAGGGAGGGAGGA	100	destroyed	19.1	19
11	RNA	GGACGGACGGACGGA	100	destroyed	11	13
12	RNA	GGUUGGUUGGUUGGA	100	destroyed	17	14
13	RNA	GGCACGGCACGGCACGGA	191.6	destroyed	141.8	141.3
14	RNA	GGACAGGACAGGACAGGA	95.8	destroyed	23	14
15	RNA	GGAGCGGAGCGGAGCGGA	191.6	destroyed	181.8	120.8
16	RNA	GGAGGAGGAGG	100	stable	-	-
17	RNA	ACGGGGGGGGGGGGGGCA	100	destroyed	-	97
18	RNA	ACGGUUGGUUGGUUGGCA	194	destroyed	117.1	117
19	RNA	GCGGUUGGUUGGUUGGCG	194	stable	-	-
20	RNA	AUGGUUGGUUGGUUGGUA	194	stable	-	-
21	RNA	ACGGCCGGCCGGCCGGCA	194	destroyed	22	10
22	RNA	CAGGUUGGUUGGUUGGAC	194	stable	-	-
23	RNA	AGGUUGGUUGGUUGGA	194	destroyed	-	170
24	RNA	GGGGGGGGGGG	100	destroyed	7	6.5
25	RNA	GGCGGCGGCGG	100	stable	-	-
26	RNA	GGUGGUGGUGG	100	stable	-	-
27	RNA	AGGAGCGGAGCGGAGCGGA	100	destroyed	17	14
28	RNA	AGGCACGGCACGGCACGGA	100	destroyed	13	5
29	RNA	GCGGCACGGCACGGCACGGCG	96	destroyed	11	6
30	RNA	AGGGGGGGGGGG-GGGGGGGGGGGA	100	stable	-	-
31	RNA	AGGUGGUGGUGG-GGUGGUGGUGGA	100	stable	-	-
32	RNA	AGGCGGCGGCGG-GGCGGCGGCGGA	100	stable	-	-
33	DNA	GGAGGAGGAGG	100	stable	-	-
34	DNA	GGGGGGGGGGG	100	destroyed	2.1	2
35	DNA	GGCGGCGGCGG	100	destroyed	2.9	2
36	DNA	GGTGGTGGTGG	100	destroyed	34	33
37	RNA	GGUUGGUUGGUUGG	100	destroyed	46	45
38	RNA	GUGGUUGGUUGGUUGGUG	100	destroyed	-	100
39 (seq1)	RNA	GCGGCGGCGGAGGCA	100	stable	-	-
40 (seq2)	RNA	GGCGGCGGCAGUGGCGGCGG	100	destroyed	7	5
41 (seq3)	RNA	AGGUGGCGGCGGC	100	stable	-	-
42	DNA	GCGGCGGCGGAGGCA	100	stable	-	-
43	DNA	GGCGGCGGCAGTGGCGGCGG	100	destroyed	9	8
44	DNA	AGGTGGCGGCGGC	100	stable	-	-

We performed two sets of MD simulations in NpT ensemble and periodic boundary conditions. The first set was based on the Amber ff99-ILDN-Bsc0 force field (Appendix B.1), and the second set employed the polarizable AMOEBA force field (Appendix B.2). The purpose and scope of both sets were different. With the classical Amber ff99-ILDN-Bsc0 force field, we wanted to examine the propensity of quadruplexes towards dissociation as quickly as possible. Therefore, no ions were added to the system except for those neutralizing the net charge. In this way, we prevented the (potential) stabilizing effects arising from the interactions of quadruplexes with solvent ions. The 44 systems listed in Table A1 were evaluated. The AMOEBA force field, on the other hand, being much more computationally demanding, was used only for the three *KRAS* systems (i.e., seq1, seq2 and seq3 in Table A1 and Figure 3), described in detail in Appendix B.2). In the latter simulations, we supplemented the systems with 150 mM NaCl, which—along with the more precise physical description of the simulated molecules provided by AMOEBA—enabled us to approach the dynamic behaviour of the *KRAS* GQ topologies in the physiological environment more closely.

### Appendix B.1. MD Simulations with Amber ff99-ILDN-Bsc0 Force Field

Table A1 shows the content of simulated systems, the length of each simulation and the final outcome. For *KRAS*, both DNA and RNA quadruplex analogues were studied. The final outcome was categorized as either “stable”, in which the core of a quadruplex retained most of its basic features, such as hydrogen bonding via Hoogsteen edges, the K${}^{+}$ in the GQ core, and pi-stacked guanine layers, or “destroyed”, which considered the loss of the central cation, followed by the progressive decay of the geometric features characteristic of quadruplexes. The outcome of each simulation was evaluated visually. Moreover, the time of the K${}^{+}$ escape and of the dissociation of the guanine core were established. In some of the simulations, the moment of K${}^{+}$ escape occurred after the core was assessed as being dissociated. This means that, despite the loss of a significant proportion of the Hoogsteen and pi-stacking interactions, the central cation (related to one or more nucleotides in the GQ core) was still in a configuration characteristic of quadruplexes.

### Appendix B.2. MD Simulations with AMOEBA Polarizable Force Field

We estimated the state of simulated quadruplexes by measuring the distances between the central K${}^{+}$ ion and the carbonyl oxygens of the core guanines. Figure A3–Figure A5 present these results for seq1, seq2 and seq3 *KRAS* G-quadruplexes, respectively. The average value of the K${}^{+}$–carbonyl oxygen distance during the phases of simulation when the cores of quadruplexes were considered “stable” was ∼2.89 Å. The structure was defined as “stable” when the central ion occupied the space between the G-tetrads, and none of the coordinating guanines diffused out of that ion further than 4.0 Å for longer than 5 ps. Within these arbitrarily chosen limits, all disruptions of the core were reversible, and did not lead to the release of the central ion from the core. Inlets in Figure A3–Figure A5 with label **1** show the structures of the GQ cores in the stable, mostly ordered state. Labels on inlets correspond to labels on charts, providing information on the time-step in which snapshots were taken for the visualization of the simulated quadruplexes. We observed two types of phenomena leading to the destabilization of GQ cores. The first was breaking the coordination and hydrogen bonds by one of the guanines (label **2** in Figure A3 and Figure A5). The other observed effect was the central ion leaving the GQ core (Figure A4, label **2** and Figure A5, label **3**). In both these cases, the central ion was partially replaced by Na${}^{+}$ from the solvent, which placed itself in the plane of the four guanines coordinating it, while the central K${}^{+}$ did not diffuse out of the core entrance. The core guanines remained in the quadruplex configuration while coordinating both involved cations. In the case of the seq3 quadruplex, K${}^{+}$ returned to the core in less than 300 ps, while in the seq2 case, it returned after almost 32 ns. For seq2, such a long-lasting loss of the K${}^{+}$ was related not only to the repulsion with Na${}^{+}$, but also to the deformation of the GQ core. The central and lateral loops encountered strong coupling with four Na${}^{+}$ ions through their backbone oxygens, which, enhanced by the pi-stacking of the nucleotides of both loops, led to the collapse of the central loop. That, in turn, exerted a sheering force on the guanine core, and the axis of the GQ core’s central channel deviated from its default perpendicular orientation related to the G-tetrads’ planes (Figure A6). Such a configuration made the return of K${}^{+}$ to the GQ core impossible due to steric repulsion. K${}^{+}$ could not return to the GQ core until the congestion of Na${}^{+}$ ions surrounded by the central loop had diffused out at the end of the simulation (Figure A4). Although all of the notified changes in the structures of the simulated quadruplexes were fully reversible, the above observations confirm the disruptive potential of long loops for the stability of GQ topologies. This effect was the most pronounced for the central loop.

## References

[1] Lim K.W., Amrane S., Bouaziz S., Xu W., Mu Y., Patel D.J., Phan A.T. (2009). Structure of the human telomere in K+ solution: A stable basket-type G-quadruplex with only two G-tetrad layers. J. Am. Chem. Soc..

[2] Varshney D., Spiegel J., Zyner K., Tannahill D., Balasubramanian S. (2020). The regulation and functions of DNA and RNA G-quadruplexes. Nat. Rev. Mol. Cell Biol..

[3] Lee D.S.M., Ghanem L.R., Barash Y. (2020). Integrative analysis reveals RNA G-quadruplexes in UTRs are selectively constrained and enriched for functional associations. Nat. Commun..

[4] Balasubramanian S., Hurley L.H., Neidle S. (2011). Targeting G-quadruplexes in gene promoters: A novel anticancer strategy?. Nat. Rev. Drug Discov..

[5] Fay M.M., Lyons S.M., Ivanov P. (2017). RNA G-Quadruplexes in biology: Principles and molecular mechanisms. J. Mol. Biol..

[6] Smirnov I., Shafer R.H. (2000). Effect of loop sequence and size on DNA aptamer stability. Biochemistry.

[7] Hazel P., Huppert J., Balasubramanian S., Neidle S. (2004). Loop-length-dependent folding of G-quadruplexes. J. Am. Chem. Soc..

[8] Pandey S., Agarwala P., Maiti S. (2013). Effect of loops and G-quartets on the stability of RNA G-quadruplexes. J. Phys. Chem. B.

[9] Zhang D.H., Fujimoto T., Saxena S., Yu H.Q., Miyoshi D., Sugimoto N. (2010). Monomorphic RNA G-quadruplex and polymorphic DNA G-quadruplex structures responding to cellular environmental factors. Biochemistry.

[10] Dai J., Chen D., Jones R.A., Hurley L.H., Yang D. (2006). NMR solution structure of the major G-quadruplex structure formed in the human BCL2 promoter region. Nucleic Acids Res..

[11] Karsisiotis A.I., O’Kane C., Webba da Silva M. (2013). DNA quadruplex folding formalism—A tutorial on quadruplex topologies. Methods.

[12] Kejnovská I., Stadlbauer P., Trantŕek L., Renc̆iuk D., Gajarský M., Krafc̆ík D., Palacký J., Bednár̆ová K., S̆poner J., Mergny J.-L. (2021). G-quadruplex formation by DNA sequences deficient in guanines: Two tetrad parallel quadruplexes do not fold intramolecularly. Chem. Eur. J..

[13] Largy E., Mergny J.L., Gabelica V. (2016). Role of alkali metal ions in G-quadruplex nucleic acid structure and stability. The Alkali Metal Ions: Their Role for Life.

[14] Bhattacharyya D., Mirihana Arachchilage G., Basu S. (2016). Metal cations in G-quadruplex folding and stability. Front. Chem..

[15] Bugaut A., Murat P., Balasubramanian S. (2012). An RNA hairpin to G-quadruplex conformational transition. J. Am. Chem. Soc..

[16] Risitano A., Fox K.R. (2003). Stability of intramolecular DNA quadruplexes: Comparison with DNA duplexes. Biochemistry.

[17] Mergny J.L., De Cian A., Ghelab A., Sacca B., Lacroix L. (2005). Kinetics of tetramolecular quadruplexes. Nucleic Acids Res..

[18] Joachimi A., Benz A., Hartig J.S. (2009). A comparison of DNA and RNA quadruplex structures and stabilities. Bioorg. Med. Chem..

[19] Zaccaria F., Fonseca Guerra C. (2018). RNA versus DNA G-quadruplex: The origin of increased stability. Chem. Eur. J..

[20] Neidle S. (2021). Structured waters mediate small molecule binding to G-quadruplex nucleic acids. Pharmaceuticals.

[21] Esposito V., Virgilio A., Randazzo A., Galeone A., Mayol L. (2005). A new class of DNA quadruplexes formed by oligodeoxyribonucleotides containing a 3^′^-3^′^ or 5^′^-5^′^ inversion of polarity site. Chem. Commun..

[22] Puig Lombardi E., Londoño Vallejo A. (2020). A guide to computational methods for G-quadruplex prediction. Nucleic Acids Res..

[23] Kuhrova P., Best R.B., Bottaro S., Bussi G., Sponer J., Otyepka M., Banas P. (2016). Computer folding of RNA tetraloops: Identification of key force field deficiencies. J. Chem. Theory Comput..

[24] Li N., Gao Y., Qiu F., Zhu T. (2021). Benchmark force fields for the molecular dynamic simulation of G-quadruplexes. Molecules.

[25] Haider S. (2018). Computational methods to study G-quadruplex–ligand complexes. J. Indian Inst. Sci..

[26] S̆poner J., Bussi G., Stadlbauer P., Kuhrová P., Banás̆ P., Islam B., Haider S., Neidle S., Otyepka M. (2017). Folding of guanine quadruplex molecules–funnel-like mechanism or kinetic partitioning? An overview from MD simulation studies. Biochim. Biophys. Acta. Gen. Subj..

[27] Ortiz de Luzuriaga I., Lopez X., Gil A. (2021). Learning to model G-quadruplexes: Current methods and perspectives. Annu. Rev. Biophys..

[28] Hong F., Schreck J.S., S̆ulc P. (2020). Understanding DNA interactions in crowded environments with a coarse-grained model. Nucleic Acids Res..

[29] Mulholland K., Sullivan H.J., Garner J., Cai J., Chen B., Wu C. (2020). Three-dimensional structure of RNA monomeric G-quadruplex containing ALS and FTD related G4C2 repeat and its binding with TMPyP4 probed by homology modeling based on experimental constraints and molecular dynamics simulations. ACS Chem. Neurosci..

[30] Hanwell M.D., Curtis D.E., Lonie D.C., Vandermeersch T., Zurek E., Hutchison G.R. (2012). Avogadro: An advanced semantic chemical editor, visualization, and analysis platform. J. Cheminform..

[31] Grimme S., Antony J., Krieg H. (2010). A consistent and accurate ab initio parametrization of density functional dispersion correction (DFT-D) for the 94 elements H-Pu. J. Chem. Phys..

[32] Boys S.F., Bernardi F. (1970). The calculation of small molecular interactions by the differences of separate total energies. Some procedures with reduced errors. Mol. Phys..

[33] Frisch M.J., Fox D.J. (2009). Gaussian 09, revision C.01.

[34] S̆poner J., Mládek A., S̆pac̆ková N., Cang X., Cheatham T.E., Grimme S. (2013). Relative stability of different DNA guanine quadruplex stem topologies derived using large-scale quantum-chemical computations. J. Am. Chem. Soc..

[35] Ortiz de Luzuriaga I., Elleuchi S., Jarraya K., Artacho E., López X., Gil A. (2022). Semi-empirical and linear-scaling DFT methods to characterize duplex DNA and G-quadruplexes in the presence of interacting small molecules. Phys. Chem. Chem. Phys..

[36] Lindorff-Larsen K., Piana S., Palmo K., Maragakis P., Klepeis J.L., Dror R.O., Shaw D.E. (2010). Improved side-chain torsion potentials for the Amber ff99SB protein force field. Proteins.

[37] Pérez A., Marchán I., Svozil D., Sponer J., Cheatham III T.E., Laughton C.E., Orozco M. (2007). Refinement of the AMBER force field for nucleic acids: Improving the description of *α*/*γ* conformers. Biophys. J..

[38] Jorgensen W.L., Chandrasekhar J., Madura J.D. (1983). Comparison of simple potential functions for simulating liquid water. J. Chem. Phys..

[39] Pronk S., Páll S., Schulz R., Larsson P., Bjelkmar P., Apostolov R., Shirts M.R., Shirts M.R., Smith J.C., Kasson P.M. (2013). GROMACS 4.5: A high-throughput and highly parallel open source molecular simulation toolkit. Bioinformatics.

[40] Guy A.T., Piggot T.J., Khalid S. (2012). Single-stranded DNA within nanopores: Conformational dynamics and implications for sequencing; a molecular dynamics simulation study. Biophys. J..

[41] Ando T., Skolnick J. (2010). Crowding and hydrodynamic interactions likely dominate in vivo macromolecular motion. Proc. Natl. Acad. Sci. USA.

[42] Salsbury A.M., Lemkul J.A. (2019). Molecular dynamics simulations of the c-kit1 promoter G-quadruplex: Importance of electronic polarization on stability and cooperative ion binding. J. Phys. Chem. B.

[43] Singh A., Singh N. (2015). Effect of salt concentration on the stability of heterogeneous DNA. Physica A.

[44] Rackers J.A., Wang Z., Lu C., Laury M.L., Lagardère L., Schnieders M.J., Piquemal J.P., Ren P., Ponder J.W. (2018). Tinker 8: Software tools for molecular design. J. Chem. Theory Comput..

[45] Rackers J.A., Wang Q., Liu C., Piquemal J.P., Ren P., Ponder J.W. (2017). An optimized charge penetration model for use with the AMOEBA force field. Phys. Chem. Chem. Phys..

[46] Wang Z. (2021). Tinker9: Next Generation of Tinker with GPU Support.

[47] Humphrey W., Dalke A., Schulten K. (1996). VMD: Visual molecular dynamics. J. Mol. Graph..

[48] Kawabata T. (2010). Detection of multiscale pockets on protein surfaces using mathematical morphology. Proteins Struct. Funct. Bioinf..

[49] Macke T., Case D.A. (1998). Modeling unusual nucleic acid structures. Molecular Modeling of Nucleic Acids.

[50] Salomon-Ferrer R., Case D.A., Walker R.C. (2013). An overview of the Amber biomolecular simulation package. WIREs Comput. Mol. Sci..

[51] Wang J., Cieplak P., Kollman P.A. (2000). How well does a restrained electrostatic potential (RESP) model perform in calculating conformational energies of organic and biological molecules?. J. Comput. Chem..

[52] Zgarbová M., Otyepka M., Sponer J., Mládek A., Banás̆ P., Cheatham T.E., Jurec̆ka P. (2011). Refinement of the Cornell et al. nucleic acids force field based on reference quantum chemical calculations of glycosidic torsion profiles. J. Chem. Theory Comput..

[53] Brown R.A., Case D.A. (2006). Second derivatives in generalized Born theory. J. Comput. Chem..

[54] Miglietta G., Cogoi S., Marinello J., Capranico G., Tikhomirov A.S., Shchekotikhin A., Xodo L.E. (2017). RNA G-Quadruplexes in Kirsten Ras (KRAS) oncogene as targets for small molecules inhibiting translation. J. Med. Chem..

[55] Mashima T., Matsugami A., Nishikawa F., Nishikawa S., Katahira M. (2009). Unique quadruplex structure and interaction of an RNA aptamer against bovine prion protein. Nucleic Acids Res..

[56] Ambrus A., Chen D., Dai J., Jones R.A., Yang D. (2005). Solution structure of the biologically relevant G-quadruplex element in the human c-MYC promoter. Implications for G-quadruplex stabilization. Biochemistry.

[57] Cheng M., Cheng Y., Hao J., Jia G., Zhou J., Mergny J.L., Li C. (2018). Loop permutation affects the topology and stability of G-quadruplexes. Nucleic Acids Res..

[58] Hao F., Ma Y., Guan Y. (2019). Effects of central loop length and metal ions on the thermal stability of G-quadruplexes. Molecules.

[59] Stadlbauer P., Trantírek L., Cheatham III T.E., Koc̆a J., Sponer J. (2014). Triplex intermediates in folding of human telomeric quadruplexes probed by microsecond-scale molecular dynamics simulations. Biochimie.

[60] Stefl R., Cheatham T.E., Spacková N.A., Fadrná E., Berger I., Koca J., Sponer J. (2003). Formation pathways of a guanine-quadruplex DNA revealed by molecular dynamics and thermodynamic analysis of the substates. Biophys. J..

[61] Mashimo T., Yagi H., Sannohe Y., Rajendran A., Sugiyama H. (2010). Folding pathways of human telomeric type-1 and type-2 G-quadruplex structures. J. Am. Chem. Soc..

[62] Heddi B., Martín-Pintado N., Serimbetov Z., Kari T.M.A., Phan A.T. (2016). G-quadruplexes with (4n - 1) guanines in the G-tetrad core: Formation of a G-triad–water complex and implication for small-molecule binding. Nucleic Acids Res..

[63] Singh V., Fedeles B.I., Essigmann J.M. (2015). Role of tautomerism in RNA biochemistry. RNA.

[64] Halder A., Bhattacharya S., Datta A., Bhattacharyyac D., Mitra A. (2015). The role of N7 protonation of guanine in determining the structure, stability and function of RNA base pairs. Phys. Chem. Chem. Phys..

[65] Matulis V.E., Lyakhov A.S., Gaponik P.N., Voitekhovich S.V., Ivashkevich O.A. (2003). 1,5-Diamino-1H-1,2,3,4-tetrazolium picrate: X-ray molecular and crystal structures and ab initio MO calculations. J. Mol. Struct..

[66] Niedzialek D., Urbanczyk-Lopkowska Z. (2007). New crystalline form of 7-amino-4-methylcoumarin (coumarin 120)—A polymorph with 1:1 valence tautomers. Cryst. Eng. Comm..

[67] Orłowski R., Clark J., Derr J.B., Espinoza E.M., Mayther M.F., Staszewska-Krajewska O., Winkler J.R., Jȩdrzejewska H., Szumna A., Gray H.B. (2021). Role of intramolecular hydrogen bonds in promoting electron flow through amino acid andoligopeptide conjugates. Proc. Natl. Acad. Sci. USA.

[68] Vovusha H., Amorim R.G., Scheicher R.H., Sanyal B. (2018). Controlling the orientation of nucleobases by dipole moment interaction with graphene/h-BN interfaces. RSC Adv..

[69] Yamauchi Y., Yoshizawa M., Akita M., Fujita M. (2009). Molecular recognition and self-assembly special feature: Discrete stack of an odd number of polarized aromatic compounds revealing the importance of net vs. local dipoles. Proc. Natl. Acad. Sci. USA.

[70] Kulkarni C., Bejagam K.K., Senanayak S.P., Narayan K.S., Balasubramanian S., George S.J. (2015). Dipole-moment-driven cooperative supramolecular polymerization. J. Am. Chem. Soc..

[71] Shu D., Moll W.D., Deng Z., Mao C., Guo P. (2004). Bottom-up assembly of RNA arrays and superstructures as potential parts in nanotechnology. Nano Lett..

[72] Khisamutdinov E.F., Li H., Jasinski D.L., Chen J., Fu J., Guo P. (2014). Enhancing immunomodulation on innate immunity by shape transition among RNA triangle, square and pentagon nanovehicles. Nucleic Acids Res..

[73] Li H., Lee T., Dziubla T., Pi F., Guo S., Xu J., Li C., Haque F., Liang X.J., Guo P. (2015). RNA as a stable polymer to build controllable and defined nanostructures for material and biomedical applications. Nano Today.

[74] Jasinski D., Haque F., Binzel D.W., Guo P. (2017). Advancement of the emerging field of RNA nanotechnology. ACS Nano.

[75] Ferino A., Nicoletto G., D’Este F., Zorzet S., Lago S., Richter S.N., Tikhomirov A., Shchekotikhin A., Xodo L.E. (2020). Photodynamic therapy for ras-driven cancers: Targeting G-quadruplex RNA structures with bifunctional alkyl-modified porphyrins. J. Med. Chem..

[76] Kosiol N., Juranek S., Brossart P., Heine A., Paeschke K. (2021). G-quadruplexes: A promising target for cancer therapy. Mol. Cancer.

[77] Li X., Sánchez-Ferrer A., Bagnani M., Adamcik J., Azzari P., Hao J., Song A., Liu H., Mezzenga R. (2020). Metal ions confinement defines the architecture of G-quartet, G-quadruplex fibrils and their assembly into nematic tactoids. Proc. Natl. Acad. Sci. USA.

